# Draft genome sequences of two clinical isolates of *Candida tropicalis* from Iraq

**DOI:** 10.1128/mra.01183-25

**Published:** 2025-12-19

**Authors:** Wafaa S. H. AL-Nasrawi, Ban T. Mohammed, Adnan A. Lahuf

**Affiliations:** 1 Department of Biology, College of Education for Pure Science, University of Kerbala 566625, Karbala, Iraq; 2 Department of Basic Science, College of Nursing, University of Kerbala 125663, Karbala, Iraq; 3 Plant Protection Department, Agriculture College, University of Kerbala 125663, Karbala, Iraq; Rochester Institute of Technology, Rochester, New York, USA

**Keywords:** *Candida tropicalis*, genome, antibiotic resistance, clinical isolates, Iraq

## Abstract

*Candida tropicalis* is an emerging human pathogen associated with oral candidiasis. In the current study, we report the draft genome sequences of two *C. tropicalis* isolates that were associated with multidrug resistance and outbreaks in different hospitals in Karbala province, Iraq.

## ANNOUNCEMENT

*Candida tropicalis* is an important pathogen increasingly detected in healthcare settings worldwide, where it contributes significantly to candidemia-associated mortality and clinical burden (1–3). In Iraq, reports from Karbala and Basrah hospitals confirm its regional relevance (4, 5), emphasizing the need for routine species-level identification, antifungal susceptibility testing, and improved infection-control programs (1–3).

In this study, *Candida tropicalis* was isolated from candidiasis cases involving children and older adults in Karbala province. Human oral swabs (*n* = 51) were collected with official permission from the administration of Imam Hussein Hospital. Swabs were streaked on Sabouraud dextrose agar (HiMedia, India) and incubated at 37°C for 24–48 h. Colony morphology and microscopic features were examined for preliminary identification. Subcultured isolates underwent molecular confirmation through PCR amplification of the ITS1–5.8S–ITS2 region followed by Sanger sequencing (6). Sequence identity was determined using BLASTn (version 2.16.0) (7) against the NCBI non-redundant nucleotide database with default parameters (*E* value 10, word size 11). The ITS sequences showed 97%–99% identity to documented *C. tropicalis* isolates and were deposited under accession numbers PV715407.1 and PV706983.1. Antifungal susceptibility testing of seven *Candida* isolates showed that five were susceptible to fluconazole, voriconazole, caspofungin, micafungin, amphotericin B, and flucytosine. One isolate (*C. tropicalis* A1) exhibited resistance to caspofungin and micafungin.

Draft genomes of *C. tropicalis* isolates Karbala-1-A1 and Karbala-1-B1 were generated by whole-genome sequencing at BGI (China). Genomic DNA was extracted using a magnetic bead-based protocol and prepared with the MGIEasy Universal DNA Library Prep Set. Sequencing was performed on the DNBSEQ-T7 platform, generating 150 bp paired-end reads at ~60× coverage. Raw read counts were 14,596,664 for isolate A1 and 16,119,896 for isolate B1. Reads were quality-filtered with SOAPnuke with default parameters. *De novo* assembly was conducted using SPAdes v.3.15.5 (8), and contigs were scaffolded in Geneious v.2025.2.1 (9, 10).

Genome sizes were 13,580,490 bp (A1) and 13,324,185 bp (B1), with similar GC contents (32.95% and 32.99%). Isolate B1 showed greater contiguity, reflected in higher N50 (4,146 vs 2,929 bp), lower L50 (514 vs 946), and a longer maximum contig (151,467 vs 137,256 bp). Gene prediction using Augustus (Galaxy v.3.5.0) identified 7,526 genes in A1 and 7,188 in B1; the higher number in A1 likely reflects assembly fragmentation.

Mitochondrial genome assemblies were also obtained. For A1, 431,471 reads mapped to the *C. tropicalis* mitochondrial reference genome NC_022160 (>98% identity; mean depth 1,193×). For B1, 599,706 reads mapped with >99% identity (mean depth 1,953×). Resulting assemblies were deposited under accession numbers PX071967.1 (A1) and PX071968.1 (B1).

Whole-genome sequencing provides high-resolution data for transmission tracking and antifungal-resistance profiling (11, 12). Variant analysis of the FKS1 gene—encoding the echinocandin target 1,3-β-D-glucan synthase (13, 14)—was performed by mapping reads to the reference strain (ASM633v3) in Geneious with thresholds of ≥10× coverage and ≥90% variant frequency. Four non-synonymous mutations were detected, supporting the utility of WGS-based resistance surveillance for guiding antifungal therapy (15).

## Data Availability

The genome data generated in this study are publicly available. The BioProject accession number is PRJNA1301428. The BioSample accession numbers for the two *Candida tropicalis* isolates, Karbala-1-A1 and Karbala-1-B1, are SAMN50445123 and SAMN50445262, respectively. The raw reads for these two isolates are available at ENA/SRA under accession numbers SRR36094885 and SRR36102999, respectively. The Whole-Genome Shotgun projects for these isolates have been deposited at DDBJ/ENA/GenBank under accession numbers JBRALD000000000.1 and JBRALE000000000.1, respectively. All records can be accessed through the NCBI database at https://www.ncbi.nlm.nih.gov.
